# Prospective cohort study of the circadian rhythm pattern in allogeneic sibling donors undergoing standard granulocyte colony-stimulating factor mobilization

**DOI:** 10.1186/scrt180

**Published:** 2013-03-20

**Authors:** Patricia A Shi, Luis M Isola, Janice L Gabrilove, Erin L Moshier, James H Godbold, Lorraine K Miller, Paul S Frenette

**Affiliations:** 1 Tisch Cancer Institute, Mount Sinai School of Medicine, 1190 5th Avenue, New York, NY, 10029, USA; 2 Department of Preventive Medicine, Mount Sinai School of Medicine, 1 Gustave L. Levy Place, New York, NY, 10029, USA; 3 Ruth L. and David S. Gottesman Institute for Stem Cell Biology and Regenerative Medicine, Albert Einstein College of Medicine, 1301 Morris Park Avenue, Bronx, NY, 10461, USA

**Keywords:** Antigens, CD34, Antigens, CD38, Circadian rhythm, Granulocyte-colony stimulating factor, Hematopoietic progenitor cells, Hematopoietic stem cell mobilization

## Abstract

**Introduction:**

Prior *in vivo* murine studies suggest circadian oscillations for hematopoietic stem cell release, which are maintained following administration of granulocyte colony-stimulating factor (G-CSF) or plerixafor. Furthermore, retrospective data analysis of healthy donors who underwent G-CSF-induced mobilization demonstrated significantly increased CD34^+^ cell yields when collected in the afternoon compared with the morning.

**Methods:**

A prospective study was conducted to directly examine the number of peripheral blood CD34^+^ and CD34^+^CD38^–^ progenitor/stem cells at baseline and then every 6 hours for 24 hours on days 4 to 5 of G-CSF (10 μg/kg/day in the morning) mobilization in 11 allogeneic donors. Data were analyzed using mixed-model analysis of repeated measures.

**Results:**

Whereas we observed a significant increase in CD34^+^ cell counts toward the evening, counts were then sustained on the morning of day 5. The correlation between CD34^+^CD38^–^ cell counts and the less defined CD34^+^ populations was weak.

**Conclusions:**

Our results suggest that the pharmacodynamic activity and timing of G-CSF may alter endogenous progenitor rhythms. Donor age, medical history, and medications may also impact circadian rhythm. Further studies should examine the circadian rhythm at the peak of G-CSF mobilization and should consider potential confounders such as the time of G-CSF administration and the age of the subjects.

## Introduction

Hematopoietic progenitor cell (HPC) mobilization using granulocyte colony-stimulating factor (G-CSF) is currently the most frequent method to obtain HPC for allogeneic transplantation. The optimal dose and schedule remains uncertain [1], but the National Marrow Donor Program uses a schedule of 10 μg/kg for 5 days, with the fifth dose ~1 hour prior to the initiation of the first leukapheresis [2]. CD34^+^ cell doses >4.5 to 5×10^6^/kg are associated with improved overall survival and reduced transplant-related mortality in the allogeneic setting [3,4]. Although obtaining this goal in a single apheresis is preferable for donor convenience and safety, only 68% of G-CSF mobilized allogeneic donors reach a CD34^+^ cell dose ≥4×10^6^/kg in a single apheresis, with a similar percentage for pegfilgrastim [5,6], and about 70% of allogeneic donors actually undergo two leukaphereses [2]. In addition, the timing of leukapheresis collection on days 5 and 6 is standardized based on staffing convenience rather than on kinetic data regarding the CD34^+^ cell number.

Based on preclinical and clinical data showing circadian oscillations of hematopoietic progenitor and stem cell release that are maintained with G-CSF or plerixafor mobilization [7-10], we conducted a single-center, prospective cohort study in allogeneic donors undergoing standard National Marrow Donor Program G-CSF mobilization (10 μg/kg/day for 5 days, administered between 06:00 and 07:00 hours), with the hypothesis that evening CD34^+^ concentrations are significantly higher than morning CD34^+^ concentrations. This study is important because hematopoietic progenitor collections typically occur in the morning due to convenience. The National Marrow Donor Program regimen was chosen because it is the most frequently used G-CSF regimen for unrelated allogeneic donors in the United States.

## Methods

This trial was a single-center, prospective cohort IRB-approved (Mount Sinai School of Medicine IRB, #08-0446) study in allogeneic sibling donors. Written and signed consent was obtained from both donors and recipients. All sibling donors for allogeneic transplants at Mount Sinai Medical Center from January 2009 to December 2011 were approached to participate. Baseline CD34^+^ concentrations prior to starting G-CSF were assessed with two separately collected samples. Donors were admitted the evening of day 3 of G-CSF to the Clinical Research Center at Mount Sinai and stayed through two nights until the morning of day 5 of G-CSF mobilization, when they were discharged to the apheresis center to start leukapheresis. All donors reported normal sleep habits on specific review of symptoms and, except for one from Wisconsin, lived in the New York City area and had no history of travel within the past month outside their normal time zone.

**Figure 1:**
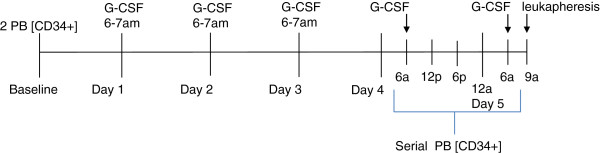
**Clinical study design.** Blood samples for the number of CD34^+^ cells ([CD34^+^]) / CD34^+^CD38^–^ cells ([CD34^+^CD38^–^]) at 06:00 hours were drawn immediately prior to granulocyte colony-stimulating factor (G-CSF) administration on days 4 and 5. PB, peripheral blood.

Starting at 06:00 hours on day 4, prior to G-CSF, we measured the number of CD34^+^ cells ([CD34^+^]) and CD34^+^CD38^–^ cells ([CD34^+^CD38^–^]) in peripheral blood (PB) every 6 hours for 24 hours (Figure 1). Blood was drawn through a 20-gauge angiocath attached to a medlock placed upon admission, to avoid distress from multiple needlesticks. Samples were drawn after discard of 2 ml PB, and were run using a standard technique (Stem-Kit®, CD38 Ab; Beckman Coulter, Brea, CA, USA) in the clinical flow cytometry laboratory. Approximately three blood volumes were processed on the first day of collection in all donors.

Statistical analysis was performed using the following software: Excel® 2007 (Microsoft Corporation, Redmond, WA. USA), SPSS Statistics® 18 (IBM Corporation, Armonk, NY, USA), and SAS version 9.2 (SAS Institute Corp., Cary, NY, USA).

## Results

Eleven healthy allogeneic sibling donors were recruited into this study (Table 1). All participants were over 45 years of age except for three donors (ages 20, 29, and 30) and were 10/10 HLA matched except for one donor, who was an 8/10 HLA match (mismatched at DRB1 and DQB1). Stem cell mobilization was successfully completed in all subjects, with all measurements completed except for one donor, in whom the 18:00 hours blood sample was drawn but misplaced. All recipients had a Karnofsky score of 90 or 100, received graft-versus-host-disease prophylaxis with tacrolimus/methotrexate or cyclosporine/mycophenolate mofetil, and have either died or been followed for at least 1 year (Table 2).

**Table 1:** Summary of allogeneic sibling donor characteristics

**Gender**	**Age (years)**	**Medical diseases (number of patients)**
6 females	Average: 48	Asthma: 1
5 males	Median: 52	Coronary artery disease: 1
	Range: 20 to 63	Diabetes mellitus type II: 2
		Hyperlipidemia: 3
		Hypertension: 3

**Table 2:** Summary of recipient characteristics

**Gender**	**Age (years)**	**Hematologic malignancy (number of patients)**	**Conditioning regimen**	**Acute GVHD**	**Chronic GVHD**	**CD34 dose**^**a **^**per recipient kg (×10**^**6**^**)**
5 females	Average: 46	Acute lymphoblastic leukemia: 1	Myeloablative: 2	Grade I: 1	Limited: 3	Average: 8.1
6 males	Median: 56	Acute myelogenous leukemia: 3	Nonmyeloablative: 1	Grade II: 4	None: 8	Median: 9.0
	Range: 23 to 60	Hodgkin disease: 1	Reduced intensity: 8	Grade III: 2		Range: 3.6 to 10.0
		Myelodyplastic syndrome: 2		None: 4		
		Non-Hodgkin lymphoma: 3				
		Plasma cell leukemia: 1				

GVHD, graft-versus-host disease. ^a^In seven patients, the infused amount was less than the amount collected from the donor (remainder frozen).

**Figure 2:**
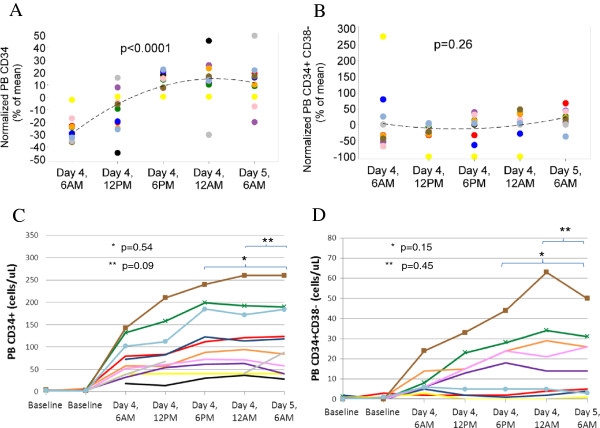
**Study outcome data.** (**A**) Normalized peripheral blood (PB) concentration of CD34^+^ cells ([CD34^+^]). (**B**) Normalized PB concentration of CD34^+^CD38^–^ cells ([CD34^+^CD38^–^]). (**C**) Absolute PB [CD34^+^]. (**D**) Absolute PB [CD34^+^CD38^-^]. *n* = 11 patients. Each donor is identified with the same color in all graphs. For (**A**) and (**B**), each dot represents an individual donor’s percentage change from their PB mean. A mixed model was used to estimate the quadratic trend in normalized values over time, assuming an autoregressive order 1 correlation structure. The three youngest donors have time point markers added to their lines. *P* values were obtained using paired *t* testing.

Average absolute baseline PB [CD34^+^] and [CD34^+^CD38^–^] were 3/μl (range 2 to 7/μl) and 0.6/μl (range 0 to 2/μl), respectively. Owing to the wide variation in the absolute number of HPCs mobilized in response to G-CSF among individual donors, each donor’s PB [CD34^+^] and [CD34^+^CD38^–^] was normalized as a percentage of that donor’s mean of day 4 through day 5 values [7] (Figure 2A,B). There was a very significant rise over day 4 in the PB [CD34^+^] concentration (*P* <0.0001), consistent with the maintenance of circadian oscillations. However, there was no significant difference in PB [CD34^+^] and [CD34^+^CD38^–^] comparing the day 4 evening (18:00 or 00:00 hours) with the day 5 morning (06:00 hours) value. Analyzing absolute concentrations, there were also no significant differences between day 4 evening and day 5 morning PB [CD34^+^] or [CD34^+^CD38] values (Figure 2C,D). The three younger donors aged <45, however, had the highest [CD34^+^] mobilization curves and exhibited or maintained their peak [CD34^+^CD38^–^] at day 4 midnight.

**Figure 3:**
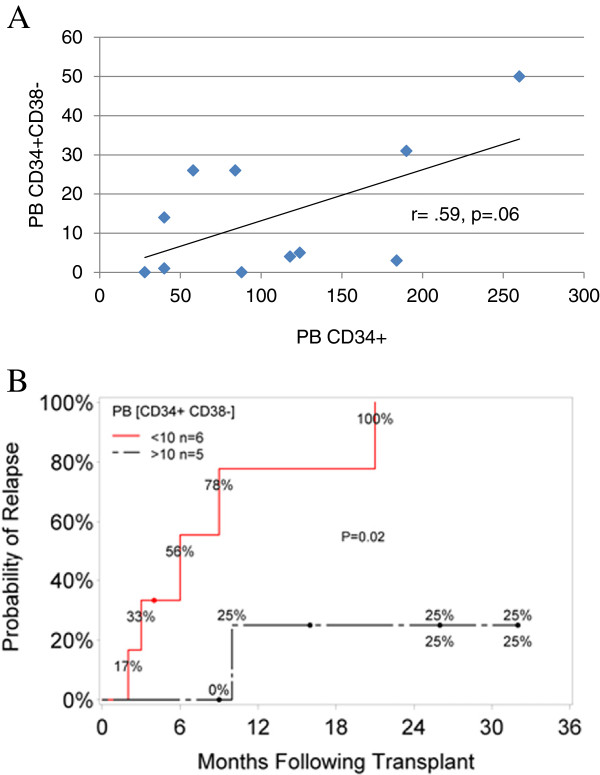
**Hypothesis-generating data.** (**A**) Correlation between day 5 peripheral blood (PB) concentration of CD34^+^ cells ([CD34^+^]) and concentration of CD34^+^CD38^–^ cells ([CD34^+^CD38^–^]) (in cells/μl). The *r* value was obtained using Pearson correlation. (**B**) Kaplan–Meier probability of relapse based on day 5 PB [CD34^+^CD38^–^] <10/μl or >10/μl. Probabilities of relapse were estimated using the Kaplan–Meier product limit method with comparison between groups evaluated by the log-rank statistic.

The correlation between the 06:00 hours PB [CD34^+^] and [CD34^+^CD38^–^] was weaker than expected (Figure 3A) and the utility of using PB CD34^+^CD38^–^ compared with CD34^+^ alone to determine the optimal time for collection is worth exploring given the correlation between CD34^+^CD38^–^ graft content and early engraftment in the allogeneic setting [11,12] and long-term neutrophil and platelet reconstitution in the autologous transplant setting [13,14].

*Post-hoc* Kaplan–Meier analysis (although hypothesis-forming only) showed a significant correlation (*P* = 0.02) between PB [CD34^+^CD38^–^] and relapse (Figure 3B), of interest since donor CD34^+^CD38^–^ stem cells may compete against leukemic CD34^+^CD38^–^ stem cells that influence relapse [15,16]. Although a product CD34^+^CD38^–^ count was not performed, the correlation coefficient between product CD34^+^ count/donor weight and 06:00 hours PB [CD34^+^] was 0.86.

## Discussion

In previous human studies, there was an average >2-fold difference in baseline (nonmobilized) circadian variation in HPCs [7,9,10]. In this prospective cohort study of 11 G-CSF mobilized allogeneic sibling donors, there was a highly significant rise in PB [CD34^+^] and [CD34^+^CD38^–^] throughout day 4 (*P* <0.0001), but no reductions in the morning of day 5, as might be expected with a circadian rhythm pattern.

The expected trough on the morning of day 5 may be mitigated by the effect of G-CSF. Previous pharmacodynamic studies show that the peak of G-CSF-induced mobilization occurs on days 5 and 6 [17,18]. The significant difference in absolute mean 06:00 hours HPC concentrations (*P* = 0.004 for [CD34^+^], *P* = 0.037 for [CD34^+^CD38^–^], paired *t* tests) between day 4 and day 5 is consistent with a previous report [19].

On the contrary, with a G-CSF effect alone one might expect the day 5 06:00 hours [CD34^+^] / [CD34^+^CD38^–^] to be significantly higher than the day 4 evening values; thus the absence of this finding is consistent with a circadian rhythm. Ethical considerations precluded postponing leukapheresis to evaluate the day 5 to day 6 PB HPC concentrations, which might effectively address the conundrum of dissecting the circadian rhythm from the G-CSF pharmacodynamic effect, which plateaus during this time period [19].

The timing of G-CSF administration in the morning may have overridden endogenous evening peak HPC counts except in the youngest donors. In a study of serial administration of a 4-day G-CSF course at different hours of the day [20], the peak of the CD34^+^ count on the fifth day occurred within several hours of the G-CSF administration time on the previous 4 days (Georg Bjarnason, personal communication). On day 5 of daily morning G-CSF administration, there was also a nonsignificant trend in 10 healthy volunteers towards a peak CD34^+^ count 2 hours after the G-CSF dose [19]. In the mouse model, where circadian rhythms were maintained with G-CSF mobilization [9], human G-CSF was injected subcutaneously at 125 μg/kg every 12 hours for 4 days, and blood collection was always timed at 3 hours after the last dose of G-CSF. Perhaps the steadier serum levels of G-CSF obtained with every 12-hour dosing [21] is important for maintenance of the circadian rhythm, given G-CSF pharmacokinetics of a maximum serum concentration after subcutaneous administration for 2 to 8 hours and a half-life of ~3.5 hours.

The circadian pattern of stem cell egress may be altered in older people, as it is notable that the three youngest donors had the highest [CD34^+^] mobilization, and reached or maintained their peak [CD34^+^CD38^–^] at day 4 midnight. Older patients do not mobilize hematopoietic progenitors as well as young subjects [22] and previous studies finding an endogenous circadian rhythm used as their subjects young mice aged 7 to 8 weeks old [9] or healthy volunteers with mostly an age range in their 20s and 30s [7-10].

Donor ailments or medications may have affected their circadian rhythm, as stem cell mobilization may be affected by diabetes [23] or by drugs affecting sympathetic tone [24,25]. The two diabetic patients had relatively low CD34^+^ mobilization, while the asthmatic donor on the β_2_-adrenergic agonist albuterol (and α-adrenergic agonist pseudoephedrine) had the highest mobilization.

Finally, the circadian peak may have occurred between 18:00 and 00:00 hours and was thus missed. Previously reported ~2-fold differences in PB [CD34^+^] / [CD34^+^CD38^–^] were seen at 20:00 hours compared with 08:00 hours collection times [9]. Previous mouse data suggest that the circadian HPC concentrations can fluctuate significantly over 4-hour intervals [8].

The relatively poor correlation of [CD34^+^CD38^–^] with [CD34^+^] and its possible prognostic importance suggest that [CD34^+^CD38^–^] might be further explored as a determinant of the optimal time for collection.

## Conclusion

This is the first prospective cohort study based on preclinical mouse as well as retrospective clinical data to examine whether circadian oscillations to hematopoietic stem cell release are maintained with G-CSF (or plerixafor) mobilization. Such data are important because a simple change of collection time to coincide with the peak of circadian egress might theoretically optimize the number of HPC collected. Our data suggest that endogenous progenitor rhythms may be altered by pharmacodynamic effect and the timing of G-CSF, and also donor age, medical history, and medications. These findings have important implications for future study design using G-CSF or possibly other mobilizing agents to study HPC biology.

## Abbreviations

G-CSF: granulocyte colony-stimulating factor; HPC: hematopoietic progenitor cell; PB: peripheral blood

## Competing interests

JLG (a study co-investigator) is a named inventor of G-CSF. This invention was patented by Memorial Sloan Kettering Cancer Center and licensed to Amgen. JLG receives a portion of the royalty payments that Memorial Sloan Kettering Cancer Center receives for this patent. G-CSF is US Food and Drug Administration approved and is used clinically as part of the standard of care for the normal mobilization and collection of stem cells in the context of peripheral blood stem cell transplants. There remaining authors declare they have no competing interests.

## Authors’ contributions

PAS conceived and designed the study, acquired and analyzed data, and wrote the manuscript. LMI helped design the study, acquired and analyzed data, and edited the manuscript. JLG edited the manuscript. ELM analyzed data. JHG analyzed data. LKM helped design the study and acquired data. PSF conceived and designed the study, and wrote the manuscript. All authors read and approved the final manuscript.
